# A data-driven remote sensing approach for VMS mineralization mapping: Integrating Sentinel-2 imagery with geology data in the Asmara Belt, Eritrea

**DOI:** 10.1371/journal.pone.0353934

**Published:** 2026-07-24

**Authors:** Segen M. Habtemichael, Woldegabriel Genzebu, Selamawit T. Ghebremicael, Yacob T. Tesfaldet

**Affiliations:** 1 Department of Earth Sciences, Mai-Nefhi College of Science, Asmara, Eritrea; 2 Alpha Exploration, Asmara, Eritrea; Khalifa University, UNITED ARAB EMIRATES

## Abstract

Mineral resources play a critical role in the sustainable economic development of countries. In Eritrea, conventional mineral exploration and geological mapping methods are expensive, time-consuming, and in some inaccessible areas, difficult to implement. Advances in remote sensing and data-driven analytical techniques now provide efficient alternatives for mineral exploration. This research applies a remote sensing and data-driven approach, combining multispectral Sentinel-2 imagery with field geological data, to identify volcanogenic massive sulfide (VMS) deposits and map lithology in the arid Asmara mineralized belt of Eritrea. Image processing techniques, including band ratios and Feature Oriented Principal Components Selection (FPCS), were combined with supervised classification algorithms such as Maximum Likelihood, Minimum Distance, and Spectral Angle Mapper to derive geological classes. Field data, including rock samples and GPS locations of known VMS gossans, were integrated for model training, thin-section validation, and performance assessment. The results demonstrate that hydrothermal alteration zones associated with VMS deposits, expressed as oxidized gossans, were effectively distinguished from widespread but unmineralized hematitic laterites using the Chica-Olma ratio method and supervised classification algorithms. The derived lithological and alteration maps show strong agreement with existing geological maps, and the locations of known VMS deposits, underscoring the potential of combining Sentinel-2 imagery and geological field data for mineral exploration in the Arabian–Nubian Shield.

## 1. Introduction

Volcanogenic massive sulfide (VMS) deposits represent globally significant polymetallic ore systems that supply substantial proportions of the world’s copper, zinc, lead, gold, and silver [1,2]. These deposits typically form in submarine volcanic environments through the discharge of hot, metal-rich hydrothermal fluids at or near the seafloor, producing lenses of polymetallic massive sulfides [3,4]. Their identification and delineation from surface expressions, particularly gossans and hydrothermal alteration halos, remain central challenges in mineral exploration, especially in remote and geologically under-explored terranes such as the Arabian-Nubian Shield (ANS) of northeastern Africa and Arabia [5,6].

The spectroscopic basis for remote sensing of hydrothermal alteration minerals is well established. Diagnostic absorption features of hydroxyl-bearing minerals, including clay minerals (kaolinite, illite, montmorillonite), micas (muscovite, sericite), and carbonate-bearing alteration products, occur predominantly in the short-wave infrared (SWIR) spectral region (1.4–2.5 μm), while iron oxide minerals such as goethite, hematite, and jarosite exhibit characteristic absorption features in the visible and near-infrared (VNIR) portions of the spectrum [7–9]. These spectral properties form the physical basis for band ratio and principal component analysis (PCA) techniques that are widely applied in geological remote sensing. The USGS Spectral Library provides the reference spectra for these diagnostic minerals, enabling robust calibration of remote sensing outputs against known mineral assemblages [9].

The application of remote sensing to geological mapping and alteration detection has evolved considerably over the past four decades. Early studies employed Landsat TM/ETM+ band ratios and the Crosta PCA technique to map alteration minerals at regional scales [10]. Subsequent advances leveraged ASTER’s enhanced SWIR coverage to delineate argillic, phyllic, and propylitic alteration zones with greater mineral specificity [11–13]. More recently, multi-sensor data fusion approaches combining Sentinel-2, ASTER, and Landsat imagery have demonstrated improved mineral discrimination [14], while machine learning algorithms including Random Forest, support vector machines, and deep learning approaches have substantially advanced mineral prospectivity mapping [15,16]. Sentinel-2, launched in 2015, offers a strategic combination of band placement in the SWIR region, 10–20 m spatial resolution, and a free and open data policy, making it a highly practical platform for reconnaissance-scale alteration mapping. However, Sentinel-2 is a multispectral rather than hyperspectral sensor; its band width limitations mean that mineral discrimination is less specific than that achievable with hyperspectral instruments such as PRISMA or EnMAP. This constraint must be borne in mind when interpreting outputs, and classification results require integration with field data for reliable interpretation.

Gossans are near-surface, iron oxide-rich zones that overlie sulfide-bearing ore deposits, formed through the oxidation and leaching of sulfides [17]. VMS deposits, commonly associated with hydrothermal fluids hosted in volcanic rocks [2], exhibit hydrothermal alterations in their host rocks. Hydrothermal alteration occurs when pre-existing minerals respond to physical and chemical conditions different from those under which they originally formed, primarily due to the action of hydrothermal fluids [18]. These deposits typically form in submarine volcanic environments as lenses of polymetallic massive sulfides at or near the seafloor, resulting from the discharge of hot, metal-rich hydrothermal fluids [3,19]. Gossans and hydrothermal alteration zones are the primary surface-observable exploration indicators for VMS deposits; their spectral detectability at SWIR and VNIR wavelengths makes them particularly suitable targets for multispectral remote sensing [5,20].

In Eritrea, identifying gossans, surface expressions of VMS polymetallic (Cu, Pb, Zn, Au) mineralization, is crucial for efficient exploration and exploitation of these resources, as such deposits are relatively common in the region [21]. Traditional geological and mineral exploration methods are often costly in terms of time, funding, and skilled labor [22]. These high costs can significantly impact the economic viability of mining operations. Remote sensing offers a cost-effective alternative by enabling the identification of hydrothermally altered zones and gossans associated with mineral deposits, thereby reducing fieldwork expenses and accelerating exploration timelines [23]. Remote sensing has proven invaluable for geological mapping and mineral exploration, particularly in arid regions where hydrothermal alteration zones are prominent [5,20,24,25]. Addressing this gap has both scientific significance and practical importance for the sustainable economic development of Eritrea. While remote sensing and Geographic Information System (GIS) techniques have been applied in previous studies in Eritrea and the broader ANS region [5,6,26–29], there remains a significant gap in published research regarding the systematic application of contemporary multispectral platforms such as Sentinel-2 to mineral exploration in the Asmara Mineralized Belt.

This study addresses a significant gap in the application of remote sensing for mineral exploration in Eritrea by applying band ratio, Feature-Oriented Principal Component Selection (FPCS), and supervised classification image analysis techniques to Sentinel-2 multispectral data. The results are compared with detailed geological maps, and the locations of known VMS deposits to evaluate the potential of multispectral datasets for identifying gossans associated with VMS mineralization in the Asmara Mineralized Belt. Specifically, the study seeks to: (1) map hydroxyl-bearing alteration minerals and iron oxides from Sentinel-2 data using band ratio and FPCS methods; (2) derive lithological maps using supervised classifiers (Maximum Likelihood, Minimum Distance, Spectral Angle Mapper); (3) differentiate gossans from unmineralized laterites; and (4) validate spectral interpretations through petrographic thin-section analysis, and field observations.

### 1.1 Study area

**Fig 1:**
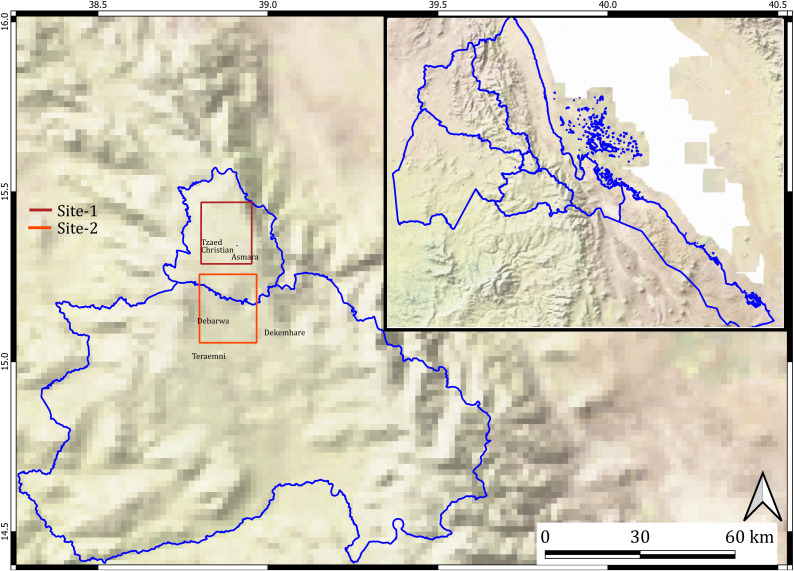
Location of the study area zoomed in from the map of Eritrea on the top right. Basemap © Natural Earth; Boundary data source: © OpenStreetMap contributors, available under the Open Database License (ODbL).

The study area is located within the Asmara Mineralized Belt [30], spanning between 38°45′4″ and 39°0′16″ longitude and 14°58′59″ and 15°28′28″ latitude (Fig 1). This mineralized deformation belt extends in an NNE–SSW direction for over 35 km and includes several mineral prospect areas, such as Embaderho and Adi Nefas to the north of Asmara, and Debarwa and Adi Rassi to the south. Approximately 15 km of the belt is covered by basalt flows, urban infrastructure, and the city of Asmara itself.

The Asmara Mineralized Belt hosts numerous mineralized zones, some of which were mined during the Italian colonial period [31,32], while others have been discovered more recently. These deposits are predominantly characterized by the presence of gossans, oxidized surface expressions of underlying mineralized bodies. Among these, the Embaderho, Adi Nefas, and Debarwa deposits have been identified as having minable potential and are currently awaiting development.

Eritrea, like many sub-Saharan countries, is situated in an arid to semi-arid region. However, the proximity of the study area to Asmara, the capital city, poses challenges for geological fieldwork due to intensive land use practices. Additionally, the rocks in the area have undergone significant weathering over time, resulting in a laterite cover that obscure much of the bedrock. This weathering, driven by a long history of humid tropical climate conditions, has further complicated direct geological investigations. Despite these challenges, the study area presents favorable conditions for remote sensing applications. The arid to semi-arid environment, combined with minimal vegetation cover, allows for clear identification of rock units, structural features, and gossan layers in multispectral datasets, such as Sentinel-2 imagery.

## 2. Geological setting

### 2.1 Regional tectonic and geological framework

The study area is situated within the Arabian-Nubian Shield (ANS), a Neoproterozoic (900–550 Ma) juvenile crustal segment that straddles the Red Sea and forms the basement of northeastern Africa and the western Arabian Peninsula [33]. The ANS was assembled through a protracted sequence of arc magmatism, arc-arc collision, and terrane accretion events during the Pan-African orogenic cycle (c. 900–600 Ma), which amalgamated oceanic island arcs, back-arc basins, and ophiolitic fragments into a coherent cratonic mass [28]. Eritrea occupies the northwestern portion of the ANS and exposes diverse Neoproterozoic lithologies including meta-volcanic and meta-sedimentary successions, syntectonic granitoids, and dismembered ophiolite sequences [30].

Regional tectonics exerted a dominant control on the distribution of mineralization in Eritrea. Major NNE–SSW-trending deformation belts developed during the final stages of the Pan-African orogeny (c. 700–600 Ma) and acted as conduits for hydrothermal fluid flow and metal precipitation [30]. These belts represent zones of intense ductile deformation, folding, and faulting that overprinted the earlier volcanic-hosted stratigraphy. The Asmara district lies within one such belt, which is characterized by upright NNE-plunging folds and associated shear zones developed under greenschist- to lower amphibolite-facies metamorphic conditions [30].

### 2.2 The Asmara Mineralized Belt

**Fig 2:**
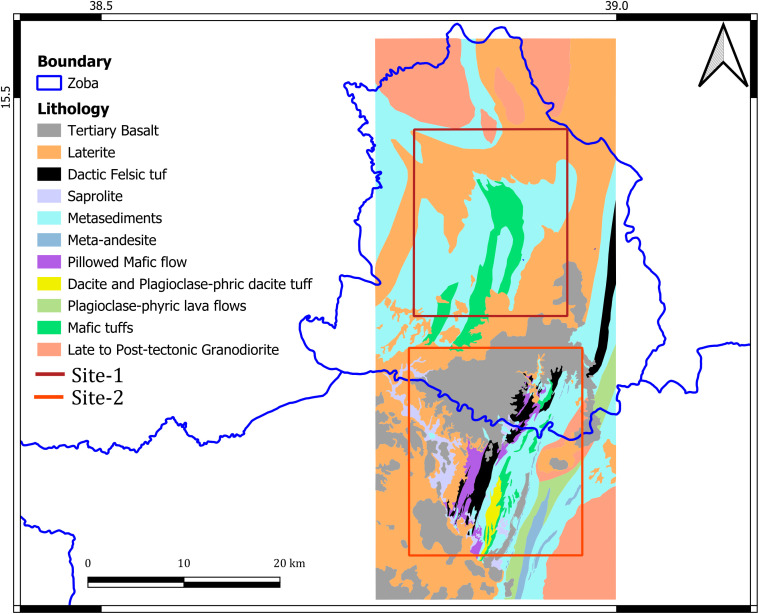
A simplified regional geology map of the Asmara Mineralized Belt. Boundary data source: © OpenStreetMap contributors, available under the Open Database License (ODbL).

The Asmara Mineralized Belt (AMB) extends for approximately 35 km in a NNE–SSW direction, flanking the eastern margin of the Asmara plateau [30]. The AMB is hosted within a Neoproterozoic bimodal volcanic sequence comprising pillowed mafic flows, mafic tuffs, flow breccias, and interbedded felsic volcanic units including quartz-phyric rhyolites, banded felsic flows, and lapilli tuffs (Fig 2). These lithologies are interpreted to represent the products of submarine volcanic activity in an intra-oceanic island arc or back-arc basin setting, consistent with the broader ANS geodynamic framework [1,30].

The volcanic sequence is intruded by syn-tectonic granodiorites and cut by a series of felsic and mafic dykes. Metamorphic grades increase southward from lower greenschist facies in the northern part of the belt to upper greenschist facies near Debarwa. Structurally, the AMB is characterized by a series of tight to isoclinal NNE-plunging folds with axial planar foliation and associated S-C fabrics indicative of sinistral shear [30]. These structural features are important exploration indicators because fold hinges and shear zone intersections are preferential sites for VMS ore body localization.

Several sub-areas within the AMB have been identified as mineral prospects, from north to south: Embaderho, Adi Nefas, Lamza, Debarwa, Adi Rassi, and Kodadu. Approximately 15 km of the central portion of the belt is covered by Tertiary basalt flows and the urban infrastructure of Asmara, limiting direct geological access to the northern and southern segments. Field studies for this investigation were therefore concentrated in two discrete sites: Site-1 (the Embaderho–Adi Nefas area to the north of Asmara) and Site-2 (the Debarwa–Kodadu area to the south).

### 2.3 VMS deposit characteristics

VMS deposits in the AMB are characterized by stratiform to stratabound lenses of massive to semi-massive pyrite, with subordinate pyrrhotite, sphalerite, chalcopyrite, galena, and gold. Cu–Zn–Pb polymetallic mineralization is the dominant style [1,31]. The deposits are hosted within the bimodal volcanic succession and are spatially associated with syn-volcanic faults and venting structures. Footwall alteration pipes composed of chlorite-sericite assemblages reflect focused hydrothermal upflow zones, while hanging-wall alteration comprising carbonate and silicification is locally preserved.

The Embaderho deposit, situated in the northern part of the AMB, represents one of the largest known VMS occurrences. It is characterized by a thick gossan cap underlain by semi-massive to massive sulfides in a felsic volcanic host. Adi Nefas, approximately 5 km to the south-southeast, is a smaller but structurally complex deposit associated with a felsite-hosted ore body and a well-developed chlorite-sericite alteration pipe. The Debarwa deposit, in the southern AMB, is hosted in a package of pillowed basalt and felsic tuffs deformed by a major fold and thrust system [30]. The Bisha VMS deposit, situated approximately 70 km west of the AMB in the Nakfa Terrane, provides a well-characterized analogue for the metallogenic potential of Eritrean Neoproterozoic terranes [1].

### 2.4 Gossans and hydrothermal alteration as exploration indicators

Gossans are the primary surface-observable exploration indicators for VMS deposits in the AMB. They develop through the complete oxidation of sulfide-bearing ore bodies and their host rocks under near-surface weathering conditions, producing iron oxide-dominated assemblages dominated by goethite, hematite, and jarosite [17]. In the Asmara region, gossans are particularly well-preserved owing to the prevailing arid to semi-arid climate, which restricts geochemical dispersion and maintains the residual oxide capping. However, widespread laterite formation driven by earlier periods of humid tropical weathering has generated extensive hematite-rich lateritic horizons that can spectrally resemble gossans. Discriminating between true gossans (indicative of VMS mineralization) and laterites (barren) is therefore a critical challenge and a primary objective of this study.

Hydrothermal alteration zones associated with VMS deposits in the AMB include: (1) a proximal chlorite ± sericite assemblage in footwall volcanic units immediately beneath the ore horizon, reflecting high-temperature (250–350 °C) acidic fluid interaction; (2) a distal propylitic halo (epidote, chlorite, carbonate) developed in more distal footwall and hanging-wall units; and (3) a hanging-wall carbonate–silica alteration zone [1,18]. The chloritic and sericitic assemblages are diagnostic because they introduce hydroxyl-bearing minerals detectable in the SWIR spectral region, while the iron-oxide-dominated gossan is detectable in the VNIR region [5,7,29]. These spectral properties justify the selection of band ratio and FPCS methods targeting these wavelength regions as the primary analytical approach in this study.

## 3. Data and methodology

### 3.1 Datasets sources and processing

Each dataset served a specific role in the investigation: Sentinel-2 multispectral imagery provided the primary remote sensing data for alteration mapping and lithological classification; QuickBird high-resolution imagery was used for visual interpretation and training sample selection; the SRTM DEM was used for topographic correction of satellite data; geological maps provided prior lithological knowledge for training and validation; rock thin sections provided petrographic evidence to validate alteration mineral assignments; and field GPS points provided spatial ground-truth for gossan occurrence verification.

The satellite images utilized in this study include Sentinel-2, QuickBird, and a Digital Elevation Model (DEM). Additionally, geological maps were integrated to support the analysis. Fieldwork was conducted to collect ground-truth data, and thin-section samples were prepared to validate interpretations derived from remote sensing imagery. A detailed list of the satellite images, datasets, and their sources is provided in Table 1.

**Table 1:** Summary of datasets used in this study, their sources, and their respective roles.

Data Type	Sensor/Product	Layers/ Bands	Date	Spatial Res.	Product Level	Source	Role in Study
Sentinel-2 MSI	T37PDS_20170112T075251/ T37PDT_20170112T075251	13	12 Jan 2017	10–60 m	Level 1C	ESA Copernicus Hub	Primary RS data for alteration mapping & classification
DEM (SRTM)	30 m SRTM	1	2000	30 m	Level 2	USGS Earth Explorer (edc.usgs.gov)	Topographic correction; terrain analysis
QuickBird	QuickBird-2	4	Various	0.6 m	Orthorectified	Asmara Mining Share Company (AMSC)	Training & validation sample selection; visual interpretation reference
Geological Maps	1:50,000	N/A	Various	N/A	Printed & digital	This study	Prior geological knowledge; training site guidance; validation
Rock Thin Sections	Petrographic slides	N/A	Field campaign	N/A	N/A	This study	Petrographic validation of alteration mineral assignments
Field GPS Data	GPS waypoints	N/A	Field campaign	Point data	N/A	This study	Ground-truth for gossan occurrences and rock sampling locations

Sentinel-2 is a European Space Agency (ESA) Earth Observation mission comprising twin satellites (Sentinel-2A and 2B) carrying the Multispectral Instrument (MSI). The MSI acquires data in 13 spectral bands ranging from the visible (443 nm) to the SWIR (2190 nm), at spatial resolutions of 10 m (bands 2, 3, 4, 8), 20 m (bands 5, 6, 7, 8A, 11, 12), and 60 m (bands 1, 9, 10). For geological remote sensing, the SWIR bands 11 (1565–1655 nm) and 12 (2100–2280 nm) are particularly valuable: band 12 covers the 2.2 μm absorption region diagnostic of Al-OH phases (sericite, kaolinite, alunite), while band 11 is sensitive to Fe-OH and Mg-OH minerals (chlorite, epidote) and also responds to ferrous iron content [14]. The combination of VNIR and SWIR bands available in Sentinel-2 enables discrimination of iron oxides (goethite, hematite) from hydroxyl-bearing alteration minerals, as demonstrated in several recent geological remote sensing studies [14,34]. Level-1C top-of-atmosphere reflectance products were used in this study, with topographic correction applied prior to analysis.

Sentinel-2 data, specifically Level-1C products, were employed in this study. These products offer varied spatial resolutions depending on the spectral band, with the dataset comprising 13 spectral bands. This expanded spectral range enhances the ability to discriminate geological features and identify hydrothermal alteration zones, which is critical for mineral exploration. The Sentinel-2 data underwent topographic correction using GRASS GIS to minimize distortions caused by terrain effects [35,36]. Subsets corresponding to the study area were extracted from the full scenes to focus the analysis.

To further refine the interpretation, vegetation and water features were masked using indices such as the Normalized Difference Vegetation Index (NDVI) and Normalized Difference Water Index (NDWI2). Threshold values were applied to create masks for vegetation and open water, enabling the isolation of geologically relevant features.

#### 3.1.1 Enhanced dataset description.

All Sentinel-2 Level-1C data were freely obtained from the European Space Agency (ESA) Copernicus Open Access Hub (https://scihub.copernicus.eu). The Level-1C products provide top-of-atmosphere (TOA) reflectance in 13 spectral bands at spatial resolutions of 10 m (bands 2, 3, 4, 8), 20 m (bands 5, 6, 7, 8A, 11, 12), and 60 m (bands 1, 9, 10). Two adjacent tiles (T37PDS and T37PDT, acquisition date 12 January 2017, orbit A008140) were used to cover the full extent of the Asmara Mineralized Belt. The SRTM Digital Elevation Model (30 m resolution) was freely downloaded from the USGS Earth Explorer platform (https://earthexplorer.usgs.gov). QuickBird high-resolution imagery (0.6 m) was provided by the Asmara Mining Share Company (AMSC) for research purposes. QuickBird imagery was used solely to guide training sample selection and visual interpretation and does not appear as a published figure in this manuscript.

#### 3.1.2 Data compliance statement.

The Sentinel-2 data used in this study are freely and openly available under the Copernicus Open Access Data Policy (https://dataspace.copernicus.eu/terms-and-conditions), which permits unrestricted use, reproduction, and redistribution of the data and derived products for any purpose, including scientific publication.“The SRTM DEM (NASA/USGS) is in the public domain with no restrictions on use. All data collection, processing, and analysis in this study were conducted in full compliance with the terms and conditions of each respective data source.

### 3.2 Image analysis and mapping

The image analysis workflow comprised two principal components: (1) Mineral information extraction using band ratios and FPCS; and (2) Land Cover Classification using supervised machine learning classifiers. These are described in the following subsections.

Individual spectral bands of Sentinel-2 datasets often fail to highlight features of interest when displayed separately. This limitation arises because the human eye can only discriminate against approximately 30 grey levels within the black-to-white range [21]. To overcome this, various image enhancement techniques are essential for visually interpreting earth materials captured by remote sensing instruments. For this study, specific Sentinel-2 spectral bands were selected based on their ability to enhance hydroxyl-bearing minerals (altered minerals) and iron oxide minerals (Table 2). These selections were guided by wavelength similarities with recommended bands from TM/ETM+ datasets [5,23,37].

**Table 2:** Band ratios of Sentinel-2 data used in this study, their geological targets, equivalent TM/ETM+ ratios, and key references. The spectroscopic rationale for each ratio is grounded in the diagnostic absorption features of the target minerals [7–9].

Sentinel-2 Band Ratio	Target Feature	Equivalent TM/ETM+ Ratio	Key Reference
11/12	Hydroxyl-bearing minerals (sericite, kaolinite); Laterite	5/7	[5,10]
11/4	Gossan (iron oxide cap)	5/4	[5]
11/2	Mafic igneous rocks	5/1	[20]
4/3	Ferric iron (Fe3+); Iron oxide discrimination	3/2	[7,8]
4/2	All iron oxides (goethite, hematite, jarosite)	3/1	[7,12]
11/8 × 4/8	Distinguish mafic from felsic rocks	5/4 × 3/4	[20]

#### 3.2.1 Mineral information extraction.

**Band ratio analysis:** Band ratios were applied to the Sentinel-2 data to reduce topographic effects and enhance the visibility of alteration minerals associated with the rocks in the study area. The specific band ratios chosen to highlight different groups of alteration minerals are listed in Table 2. Band ratios can also be combined into RGB composites to create color-enhanced images that improve visual interpretation. Several combinations of band ratios were tested, including those recommended for geological feature enhancement, such as the Sultan ratio [5], the Abrams ratio [38], as adapted for Sentinel-2), and the Chica-Olma ratio ([39], as adapted for Sentinel-2). The Chica-Olma ratio composite (bands 11/12, 11/8, 4/2 assigned to RGB) was selected as the most discriminatory for this study area as it simultaneously enhances clay-altered minerals (via the SWIR ratio 11/12), ferrous minerals (via 11/8), and total iron oxides (via 4/2) in a single false-colour display. The Abrams ratio composite (bands 4/2, 3/1, 11/8 assigned to RGB) was also evaluated and is effective for general iron oxide and hydroxyl mapping. Each composite image was carefully examined to identify those that best improved the interpretability of geological features and the most effective ratio composites were selected for further analysis.

**Principal Component Analysis (PCA)/ FPCS:** The statistical variance in multispectral images reflects the spectral response of surface materials such as rocks, soils, and vegetation. This variance is influenced by the statistical dimensionality of the image data [40]. In this study, Principal Component Analysis (PCA) was applied to Sentinel-2 data. Following the Feature-Oriented Principal Component Selection (FPCS) technique, also known as the Crosta technique [10,40], PCA was applied to specific band subsets chosen to isolate the spectral variance attributable to target mineral groups rather than to scene-wide albedo and vegetation effects. PCA can be performed on selected bands from the entire spectrum, a technique referred to as the Crosta or Feature-Oriented Principal Component Selection (FPCS) technique [10,40].

Hydroxyl-bearing minerals in arid terrains were detected in PC images when one input band was selected from the visible spectrum (e.g., Sentinel-2 bands 2, 8, 11, and 12). Similarly, iron-oxide-bearing minerals were mapped using PCA when one Short-Wave Infrared (SWIR) band was included (e.g., Sentinel-2 bands 2, 4, 8, and 11). The rationale for these band selections is as follows: for hydroxyl mapping, the inclusion of band 12 (2.2 μm) and band 11 (1.6 μm) ensures that the variance attributable to SWIR absorption by Al-OH and Fe/Mg-OH groups is captured in the high-order principal components (PC3 or PC4), where it is separated from the dominant albedo variance (PC1) and vegetation contrast (PC2) [10,41]. For iron oxide mapping, the inclusion of bands 4 (665 nm) and 2 (490 nm) targets the Fe3 + absorption edge and the broad ferric iron absorption feature in the visible region [7]. The eigenvector loadings of the relevant PC are examined to confirm that the hydroxyl or iron oxide bands carry opposite signs and high loadings, consistent with the FPCS criterion for isolating the target mineral variance [40].

#### 3.2.2 Land cover classification.

A supervised classification approach was employed, leveraging prior knowledge of the sampling area [Mather & Koch, 2004]. The classification was performed in QGIS using training sites selected from each rock type and other non-geological features present in the imagery. Three widely used algorithms namely Maximum Likelihood (ML), Minimum Distance (Min-dist), and Spectral Angle Mapper (SAM), were applied to derive a total of seven and eight geological classes for Site-1 and Site-2, respectively. Maximum Likelihood classification assumes a Gaussian distribution of spectral values within each class and assigns each pixel to the class for which the probability of membership is highest [42]. Minimum Distance classification assigns each pixel to the nearest class mean in spectral feature space, providing a computationally simple and robust comparison baseline [43]. Spectral Angle Mapper measures the spectral similarity between a pixel and each reference spectrum as the angle between their spectral vectors in n-dimensional space, making it insensitive to illumination differences [44]. All three methods were applied to both sites to enable objective comparison of classification performance across different algorithms and site conditions.

Ten Sentinel-2 Visible Near-Infrared (VNIR) and Short-Wave Infrared (SWIR) bands were used as inputs for the classification process. Specifically, bands 2, 3, 4, 5, 6, 7, 8, 8A, 11, and 12 were resampled to a common 20 m resolution and used as inputs. The 60 m bands (1, 9, 10) were excluded owing to their lower spatial resolution and their limited discriminative value for the lithological classes of interest. The results were compared to evaluate the effectiveness of the satellite imagery for geological classification. Training site selection was based on enhanced images, including decorrelation stretch, false-colour composites (e.g., Sentinel-2 bands 12-8-3), geological maps, and high-resolution 0.6-meter QuickBird imagery (Table 3). Geological boundaries, along with masks for vegetation, water bodies, and built-up areas, were overlaid on Sentinel-2 images to guide the selection process.

**Table 3:** Image classification training sample pixels for non-geological and rock classes for Site-1 and Site-2.

Site-1 Training Class	No. Training Samples	Site-2 Training Class	No. Training Samples
Granodiorite	120	Granodiorite	120
Pillowed mafic flow	240	Pillowed mafic flow	240
Laterite	420	Laterite	420
Quartz phyric felsic sub-flow	360	Quartz phyric felsic sub-flow	360
Mafic tuffs, flow breccia & lava flows	300	Tertiary basalt flow	480
Tertiary basalt flow	480	Metasediments	125
Gossan	120	Banded felsic flows, lapilli tuffs, minor mafic flows	130
		Saprolite	175
Vegetation	180	Vegetation	180
Water bodies	120	Water bodies	120
Built-up	180	Built-up	180
**Total**	**2520**	**Total**	**2530**

To ensure representativeness, training sites were chosen to capture the full variance of spectral responses within each rock type, avoiding uniform or extreme cases. According to Jensen et al. [42], the minimum number of training samples should be 10N pixels (where N is the number of input bands) to calculate covariance/variance statistics. While an absolute minimum of four training areas per class is acceptable, smaller sample sizes may compromise the reliability of standard deviation calculations [45]. Training samples were collected based on outcrop status across different areas, with additional samples for vegetation, water bodies, and built-up areas excluded after reclassifying the images into geological classes only.

The spatial distribution of training sample sites for both Site-1 and Site-2 is mapped in the results section (Section 4.2). Training sample statistics are listed in Table 3. The geological map and QuickBird imagery served as the primary references for identifying training sites: the geological map was used to constrain the broad lithological units, while QuickBird imagery enabled fine-scale visual discrimination of rock outcrops within those units. The DEM was used to exclude fluvial and alluvial deposits that might contaminate spectral signatures.

## 4. Results

### 4.1 Mineral information extraction

#### 4.1.1 Band ratio analysis.

**Fig 3:**
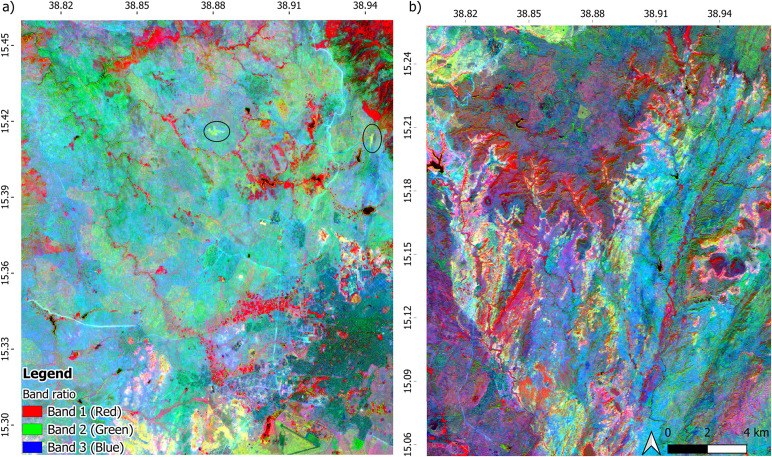
(a) Site-1; (b) Site-2: Band ratio image of Sentinel-2 (RGB = 11/12, 11/8, 4/2). This enhancement highlights hydrothermal alteration (11/12), ferrous minerals (11/8), and iron oxides (4/2). Known VMS occurrences are outlined in black polygons. Black dots indicate GPS-verified gossan points.

Band-ratio composites (Abrams’ and Chica-Olma) were generated from Sentinel-2 data to map alteration zones and to discriminate lithologies and structural features. The Abrams’ ratio composite (bands 4/2, 3/1, 11/8 in RGB) was evaluated first and confirmed the presence of iron oxide anomalies consistent with gossan distribution, as previously reported by Abdelsalam et al. (2000) for similar ANS terranes. The Chica-Olma composite (bands 11/12, 11/8, 4/2 assigned to RGB) enhanced clay-altered minerals, ferrous minerals, and iron oxides respectively (Fig 3a and 3b). In Site-1, blue tones correspond to iron-oxide concentrations within pillowed mafic flows and metasediments; green tones indicate ferrous mineral accumulations in a pillowed mafic flow; yellow-white tones correspond to lateritic cover. In Site-2, the Tertiary basalt flow displays mixed mineralogical patches (pink, blue, green). The spatial correspondence between iron oxide anomalies in the band ratio images and the known gossan locations provided the first line of evidence for the utility of this approach in the AMB. Location outlines of known VMS occurrences in Site-1 were identifiable from the band-ratio outputs. Site-2, with better rock exposure and less urban cover, showed clearer lithological discrimination than Site-1.

#### 4.1.2 Hydroxyl mapping using feature-oriented PCA (FPCS).

**Fig 4:**
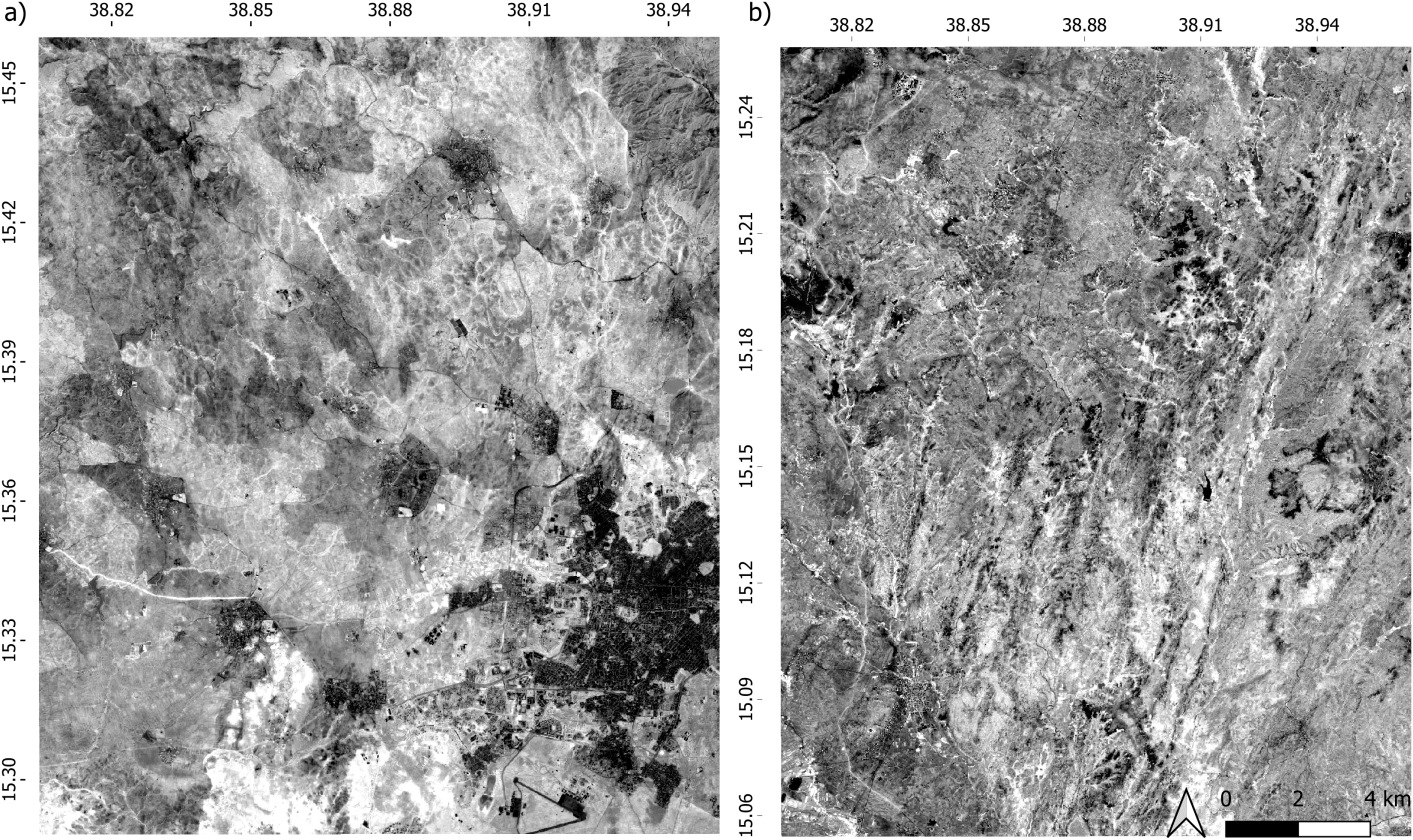
Negated PC3 (H-image), bright pixels represent the hydrothermal alterations of Site-1 (a) and Site-2 (b).

Feature-oriented principal component analysis was applied to selected Sentinel-2 bands to map hydroxyl-bearing minerals (Table 4). PC3 was identified as the hydroxyl (H) component in both sites based on eigenvector loadings for bands 11 and 12 (Site-1: band 12 = 0.54, band 11 = −0.56; Site-2: band 12 = 0.71, band 11 = −0.60). This loading pattern satisfies the FPCS criterion established by Loughlin [40] and Crosta & Moore [10]: the two SWIR bands (11 and 12) carry opposite signs and high absolute loadings, indicating that their variance contrast—primarily attributable to hydroxyl absorption at 2.2 μm—is concentrated in this PC. The negative loading of band 11 and positive loading of band 12 imply that pixels with relatively higher band 12 reflectance (i.e., weaker 2.2 μm absorption, less hydroxyl alteration) are bright in raw PC3, while pixels with stronger 2.2 μm absorption (more hydroxyl minerals) are dark. The H image was negated for display so anomalous hydroxyl concentrations appear bright (Fig 4a and 4b). PC1 primarily captured scene albedo and PC2 vegetation contrast.

**Table 4:** PCA of Sentinel-2 for mapping hydrothermal alteration of Site-1 and Site-2.

Site-1					
Input Bands	Band-2	Band-8	Band-11	Band-12	
Band Means	1221.12	2523.88	3549.44	2802.76	
SD of Bands	185.14	512.13	853.64	706.16	
Eigen vector matrix					
PC1	0.12	0.38	0.71	0.58	92.94
PC2	0.14	0.89	−0.17	−0.39	4.99
PC3	0.63	0.03	**−0.56**	**0.54**	1.57
PC4	0.76	−0.25	0.39	−0.46	0.51
Site-2					
Band Means	1116.09	2224.06	2905.12	2134.06	
SD of Bands	170.10	497.47	795.69	625.68	
Eigen vector matrix					**Eigen value (%)**
PC1	0.12	0.41	0.71	0.56	93.53
PC2	0.24	0.87	−0.27	−0.34	4.59
PC3	0.37	−0.01	**−0.60**	**0.71**	1.34
PC4	0.89	−0.29	0.23	−0.28	0.54

#### 4.1.3 Iron-oxide mapping using FPCS.

**Fig 5:**
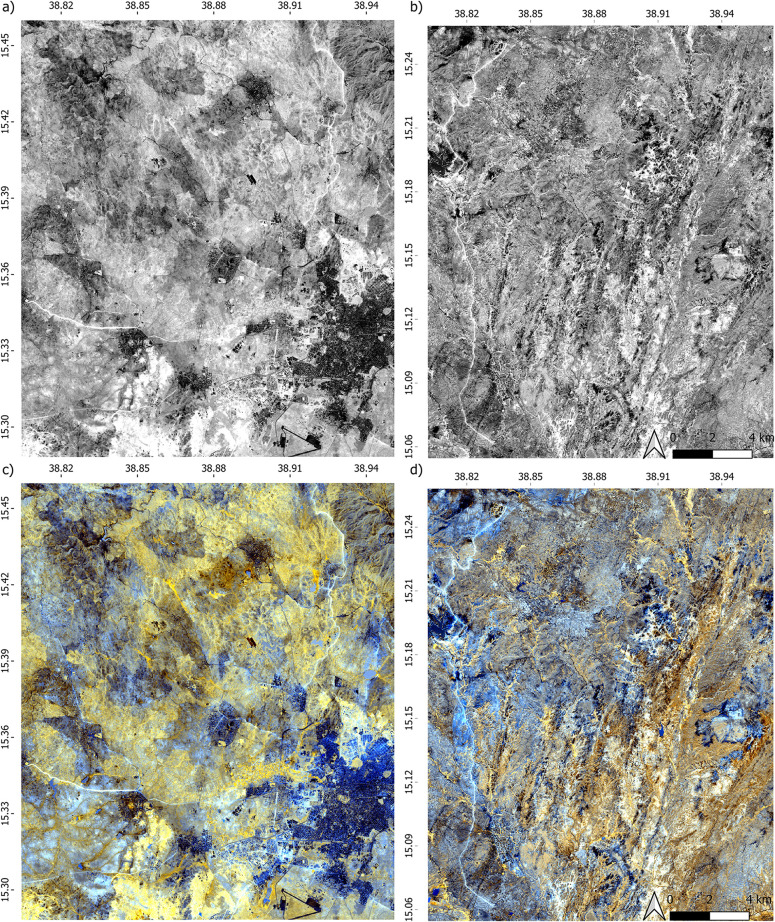
Negated PC4 (F-image), where bright pixels represent iron oxides: (a) Site 1 and (b) Site 2. Crosta’s color composite image (H-image in red, H-image + F-image in green, and F-image in blue) highlighting alteration zones in yellowish to orange-red hues: (c) Site 1 and (d) Site 2. White pixels indicate areas with both iron staining and argillization; bright reddish to orange pixels indicate argillization-dominated areas; and bright cyan to bluish pixels indicate iron-staining–dominated areas.

PCA applied to unstretched Sentinel-2 bands produced an iron-oxide (F) image (PC4) based on eigenvector loadings for band 4 and band 2 (Site-1 PC4: band 4 = −0.49, band 2 = 0.86; Site-2 PC4: band 4 = −0.43, band 2 = 0.89) (Table 5). The opposite signs of bands 4 and 2 in PC4, and their high loadings, conform to the FPCS criterion for isolating the iron oxide spectral contrast [40]. The positive loading of band 2 (490 nm) and negative loading of band 4 (665 nm) reflect the characteristic spectral slope of ferric iron minerals across the visible: Fe3 + minerals are relatively bright in the blue (band 2) compared to the red (band 4) due to the broad Fe3 + absorption feature between 600 and 900 nm [7]. Negating PC4 highlights iron-oxide-stained areas as bright pixels (Fig 5a and 5b). H and F images were combined to produce H + F composites that emphasize pixels anomalous for both hydroxyls and iron-oxides; stretched composites allowed classification of argillic versus iron-rich alteration zones (Fig 5c and 5d). White pixels indicate co-occurrence of iron staining and argillization, most likely representing true gossan sites; bright reddish-orange pixels indicate argillization-dominated alteration (sericite/chlorite zones); and bright cyan-blue pixels indicate iron-staining-dominated alteration (gossanous laterite or distal gossan). This composite approach closely follows the methodology of Crosta & Moore [10] as adapted for Sentinel-2.

**Table 5:** PCA of Sentinel-2 for mapping iron-oxide of site-1 and site-2.

Site-1					
Input Bands	Band-2	Band-4	Band-8	Band-11	
Band Means	1221.12	1857.95	2523.88	3549.44	
SD of Bands	185.14	450.79	512.13	853.64	
Eigen vector matrix					**Eigen value (%)**
PC1	0.14	0.39	0.44	0.79	91.18
PC2	0.27	0.37	0.66	−0.59	5.54
PC3	0.41	0.68	−0.61	−0.07	2.98
PC4	**0.86**	**−0.49**	0.01	0.09	0.29
Site-2					
Band Means	1116.09	1601.97	2224.00	2905.12	
SD of Bands	170.10	428.81	497.47	795.69	
Eigen vector matrix					**Eigen value (%)**
PC1	0.14	0.39	0.46	0.78	91.83
PC2	0.28	0.46	0.57	−0.62	5.76
PC3	−0.31	−0.67	0.68	−0.01	2.15
PC4	**0.89**	**−0.43**	−0.02	0.07	0.26

### 4.2 Land cover classification

Classification results for all three methods namely Maximum Likelihood (ML), Minimum Distance (Min-dist), and Spectral Angle Mapper (SAM), were produced for both Site-1 and Site-2.

#### 4.2.1 Post-classification processing.

**Fig 6:**
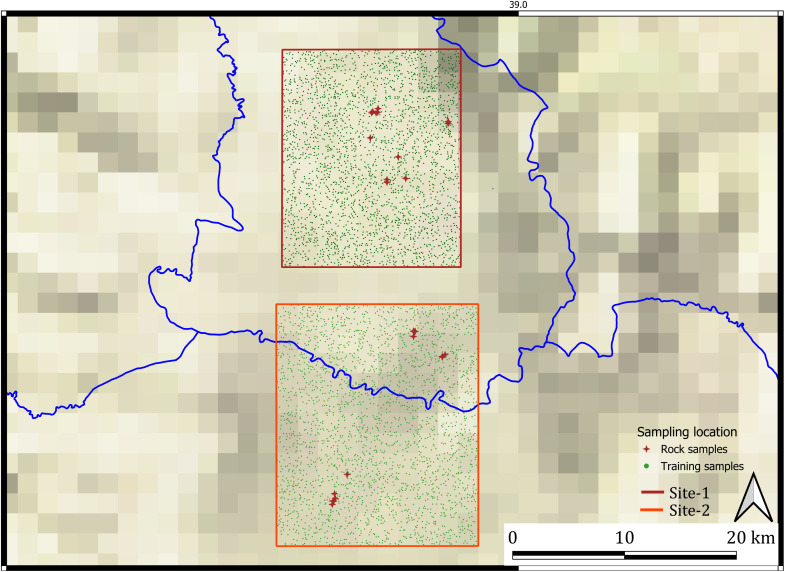
Training sample and rock sample collection points. Boundary data source: © OpenStreetMap contributors, available under the Open Database License (ODbL).

Accuracy assessment was conducted to evaluate how well pixels were classified. Testing samples were created for each geological and non-geological class using QuickBird imagery and geological maps as references (Fig 6).

Preliminary evaluations involved visually comparing the classified results to the distribution of rock units shown in geological maps. Classification accuracy was further assessed using an error matrix, which compares map information with testing samples. The error matrix includes Kappa statistics, a discrete multivariate technique for accuracy assessment. According to Congalton & Green [46], Kappa values greater than 0.80 indicate strong agreement, 0.40–0.80 indicate moderate agreement, and values below 0.40 indicate poor agreement between reference data and classified maps. The complete error matrix for each classifier and each site is provided in the supplementary material (Table S1 for Site-1 and Table S2 for Site-2 in S1 File), including per-class producer accuracy, user accuracy, overall accuracy, and Kappa coefficient. A summary of overall accuracy and Kappa coefficients for all three methods at both sites is presented in Table 6.

**Table 6:** Samples taken from Embaderho (ED), Adi-Nefas (AN), Debarwa (DB), Lamza (LM), and Kodadu (KD).

Embaderho	Adi Nefas	Debarwa	Adi Lamza	Kodadu
Meta-basalt*	Meta-basalt*	Felsic tuff*	Pyrite bearing exhalite*	Gossan
Gossan	Felsic dyke*	Gossan*	Gossan	Undifferentiated felsic flow
Rhyolite*	Barite*	Exhalite*	Felsic tuff*	Meta-andesite*
Felsic tuff	Gossan	Gossan	Exhalite	
Granodiorite*		Felsic tuff	Meta-basalt*	
Pyrite-altered mafic rock*		Barite*		
		Mafic fragmental flow		

* Samples used for thin-section analysis.

Classified images often exhibit a “salt-and-pepper” appearance due to isolated pixels. To address this, post-classification processes were applied to generalize the classes for operational use. These processes included reclassification, classification sieve, and erosion: (1) Reclassification reassigned classification classes or removed unnecessary classes; non-geological classes were assigned a value of 0 (unclassified). (2) Classification Sieve: a size threshold of 2 was applied, examining neighbouring 4 or 8 pixels to determine if a pixel belonged to a group of the same class. (3) Classification Erosion: this tool refined the classification by smoothing boundaries and eliminating small artifacts. In QGIS, these steps were applied sequentially.

#### 4.2.2 Classification accuracy and results.

**Fig 7:**
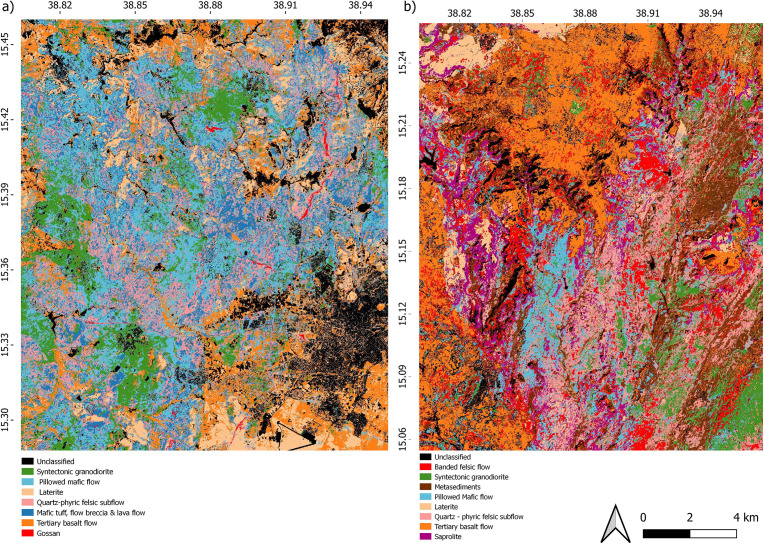
Image classifications result of Sentinel-2: (a) Min-dist classification (Site-1) (b) ML classification (Site-2).

Supervised classifications were performed using Min-dist, ML, and SAM classifiers on decorrelation-stretched and MNF-enhanced band sets. For Site-1, Min-dist performed best for lithological discrimination overall (overall accuracy: 73.34%; Kappa: 0.66), largely because the relatively simple spectral structure of Site-1 lithologies, dominated by granodiorite, laterite, basalt, and gossan, is well-suited to centroid-based classification. For Site-2, ML yielded the highest overall accuracy (73.04%; Kappa: 0.69), likely because the greater diversity and spectral overlap of lithological classes in this area better conforms to the Gaussian assumptions underlying the ML algorithm (Fig 7). Accuracy assessments indicate high producer accuracies (>98%) for gossan, Tertiary basalt flow, laterite, and syntectonic granite in Site-1; moderate to high producer accuracies (66–94%) across several classes in Site-2, with pillowed mafic flow showing the lowest producer accuracy (52.6%) (Table S1 in S1 File). Confusion between quartz-phyric and banded felsic flows, and between some granitoid and mafic units, was noted.

### 4.3 Integrated validation and accuracy assessment

Remote-sensing interpretations were validated using petrographic thin-section analysis,and verified VMS occurrences. These complementary approaches ensured that the image-based mineral mapping results were consistent with subsurface geological and mineralogical evidence.

#### 4.3.1 Field sampling and thin-section validation.

Targeted field mapping was conducted across the Asmara Belt to confirm the classification of gossan (iron-rich cap) occurrences, which are a key surface expression of VMS mineralization. Locations classified as high-probability targets by the remote sensing model were visited to verify spectral signatures against ground truth. Rock samples were collected from confirmed gossan sites and surrounding lithologies (Table 6).

**Fig 8:**
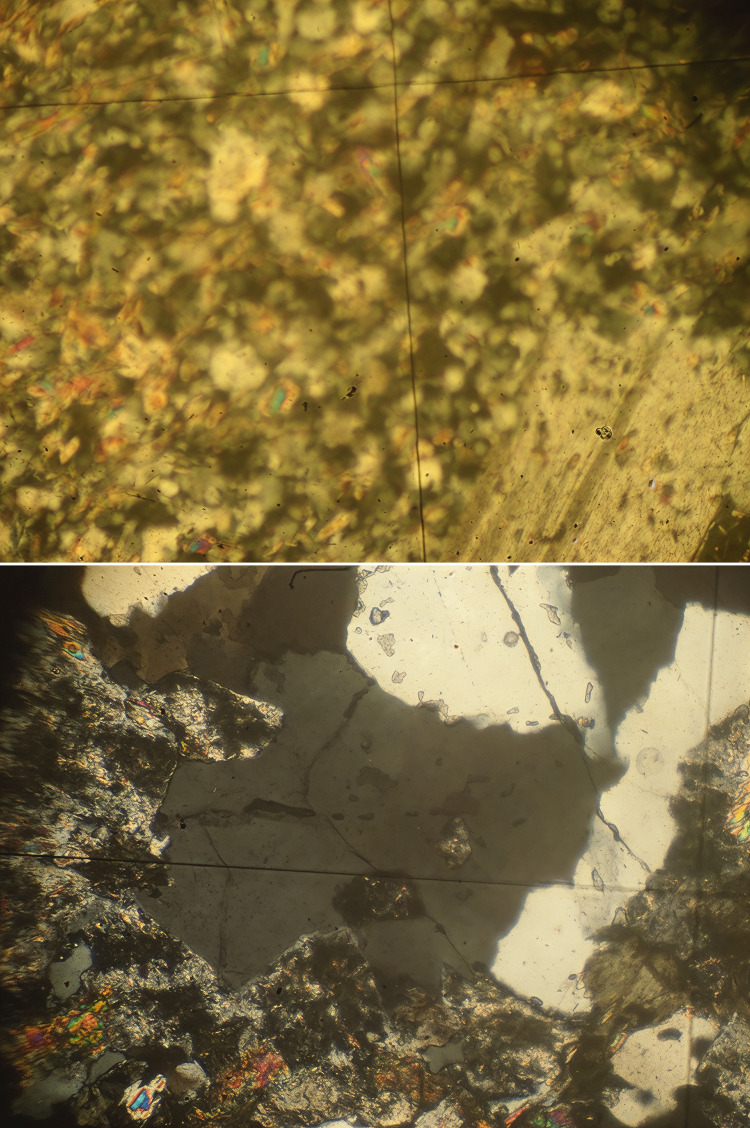
Thin-section photomicrographs of the felsic dyke (top) and syntectonic granodiorite (bottom). **Felsic Dyke**, **Syntectonic Granodiorite:** In thin sections of the Adi-Nefas felsic dyke and Embaderho syntectonic granodiorite, primary minerals, specifically plagioclase feldspar, amphibole, and ± pyroxene, are predominantly altered to sericite, epidote, and chlorite. The Adi-Nefas felsic dyke exhibits a porphyritic texture where plagioclase feldspar and quartz form the phenocrysts, while biotite and aggregates of quartz and feldspar constitute the groundmass. In contrast, the syntectonic granodiorite of the Embaderho area displays a granular texture and is dominated by recrystallized quartz.

**Fig 9:**
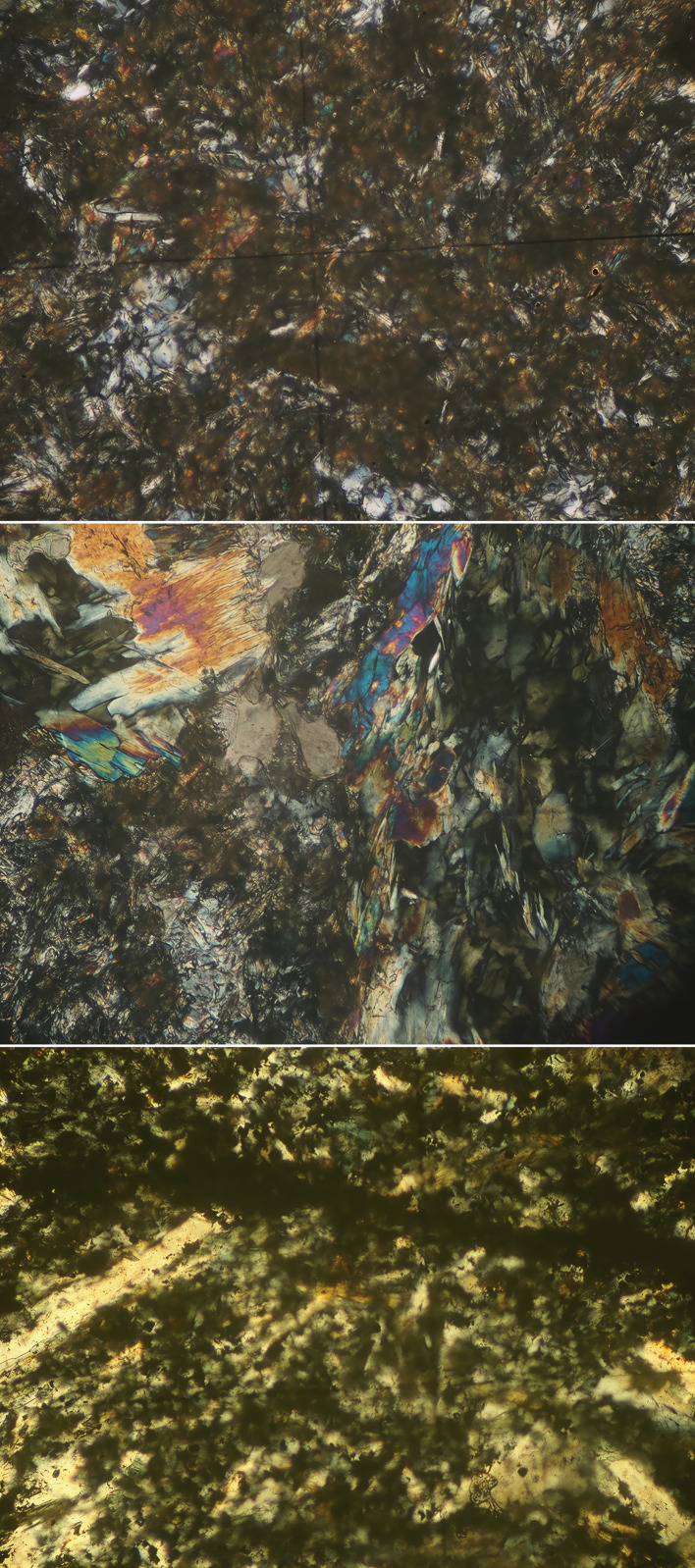
Thin-section photomicrographs of the meta-andesites. **Meta-andesites:** The meta-andesites observed in the study areas commonly contain a mineral assemblage of epidote, chlorite, plagioclase feldspar, sericite, and calcite. The Debarwa meta-andesite is medium-grained, whereas the Kodadu meta-andesite exhibits a porphyritic texture with mineral grains that are significantly larger than those in the Debarwa samples. The meta-andesite of the Adi-Lamza area also possesses a porphyritic texture, consisting primarily of plagioclase feldspar, dark spots of iron oxides, sericite, and trace amounts of quartz.

**Fig 10:**
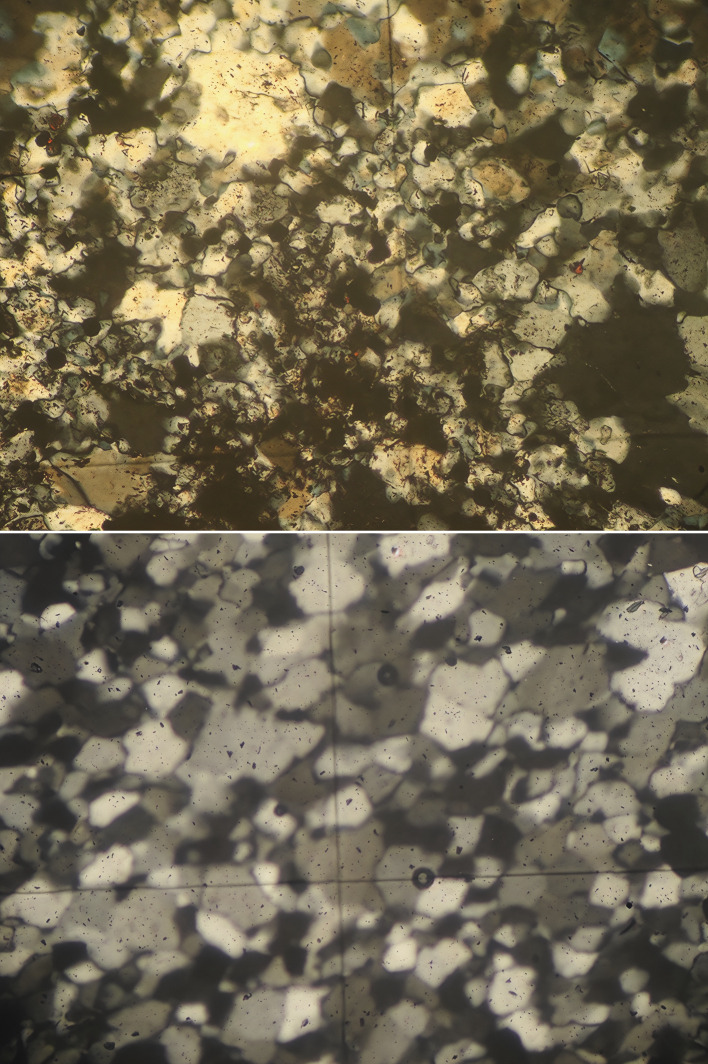
Thin-section photomicrographs of the exhalite. **Exhalite:** The minerals within the Debarwa exhalite are fine-grained and characterized by recrystallized bands of medium-grained quartz aggregates. The matrix is composed of iron oxide alongside minor epidote. Similarly, the Adi-Lamza exhalite contains fine-grained aggregates of quartz, disseminated pyrite crystals, iron oxide, and minor epidote.

**Fig 11:**
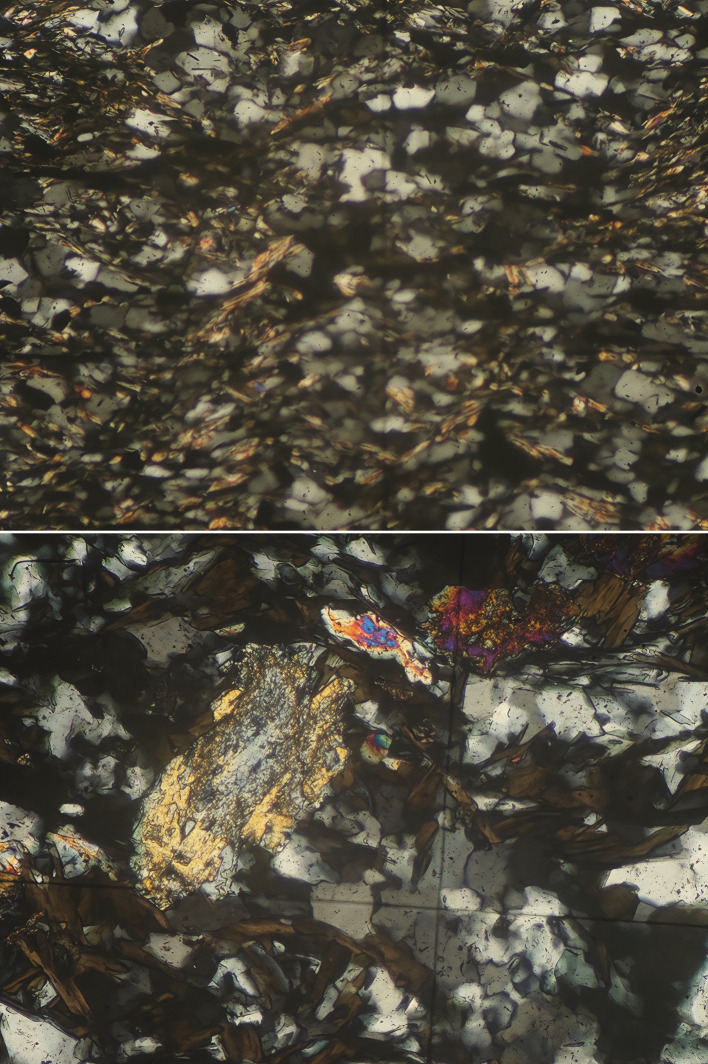
Thin-section photomicrographs of the meta-basalt. **Meta-basalt:** Thin sections of meta-basalt from the Adi-Nefas and Embaderho areas exhibit fine- to medium-grained foliated textures. The Adi-Nefas meta-basalt displays a well-developed, wavy foliation with medium- to fine-grained crystals of plagioclase feldspar, sericite, iron oxide, and minor amounts of epidote and quartz. The Embaderho meta-basalt is characterized as a medium-grained, foliated chlorite-epidote schist containing albite, sericite, and dark spots of pyrite.

**Fig 12:**
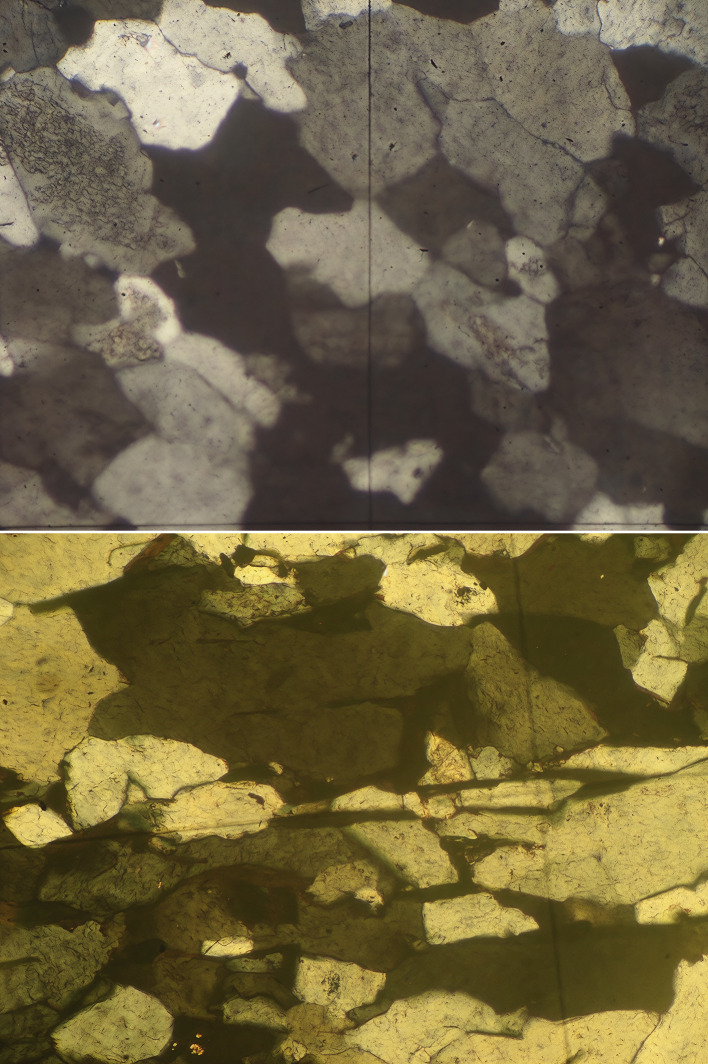
Thin-section photomicrographs of the barite. **Barite:** The Adi-Nefas barite features a poorly foliated, granular, medium-grained texture composed of chlorite and barite with dark spots of pyrite. The Debarwa barite is generally similar in character but is more specifically dominated by an assemblage of epidote and barite minerals.

**Fig 13:**
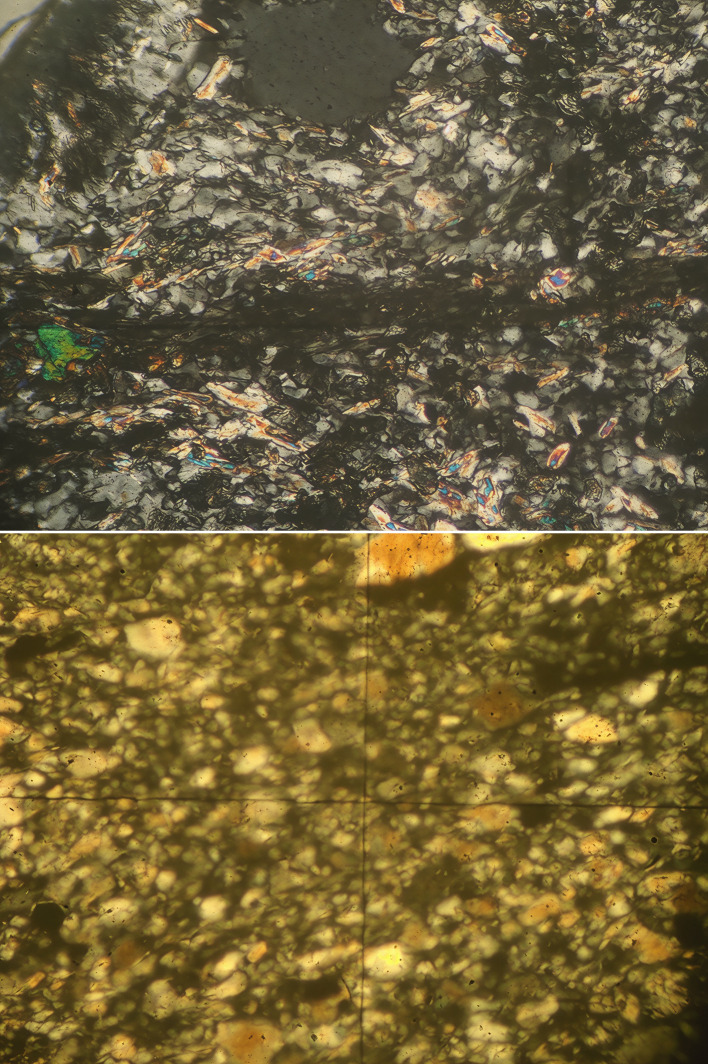
Thin-section photomicrographs of the felsic tuff. **Felsic Tuff:** The Embaderho felsic tuff is identified by a porphyritic texture featuring broken, sub-rounded quartz fragments showing wavy extinction. These fragments are set within a matrix of very fine aggregates of epidote ± quartz + feldspar + sericite. The matrix of the Adi-Lamza felsic tuff consists of fine- to medium-grained aggregates of quartz, plagioclase feldspar, epidote, and pyrite.

**Fig 14:**
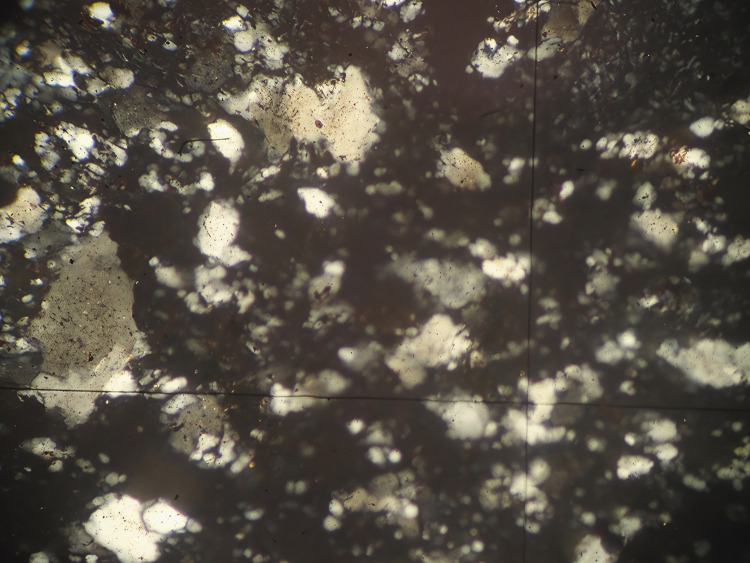
Thin-section photomicrographs of the altered felsic rock. **Altered Felsic Rock:** Additionally, the very fine- to fine-grained altered felsic rock of the Debarwa area consists of fine aggregates of quartz, iron oxide, and epidote, with microcrystalline crystallized quartz and feldspar.

Microscopic examination of representative rock samples confirmed the presence of hydrothermal alteration minerals detected through remote sensing. The thin-section analysis revealed abundant chlorite, epidote, and plagioclase feldspar, which are indicative of propylitic alteration zones (Figs 8–14). In some samples, quartz and sericite were also identified, reflecting localized silicification and sericitization, commonly associated with VMS-related hydrothermal systems. The mineral assemblages observed under cross-polarized and plane-polarized light correlate strongly with alteration patterns derived from multispectral indices and PCA-based mineral mapping. The petrographic identification of chlorite and epidote in footwall meta-volcanic units is consistent with the propylitic alteration halo expected at VMS deposits, while the occurrence of sericite in felsic volcanic units from Lamza and Debarwa indicates higher-temperature, more proximal alteration zones. These observations validate the spectral assignments of the H image (hydroxyl-rich areas) to sericite/chlorite alteration zones.

## 5. Discussion

### 5.1 Hydrothermal alteration and Iron enrichment patterns

The band-ratio and FPCS results collectively indicate spatially coherent zones of hydrothermal alteration and iron enrichment that are consistent with VMS-style mineralization [47,48]. Hydroxyl-rich anomalies identified in PC3 (H image) are interpreted as argillic/sericitic alteration halos around hydrothermal feeders or veins [10,41], while iron-oxide anomalies (PC4, F image) and H + F composites highlight oxidation and gossanous exposures commonly associated with VMS oxidized caps [49,50]. The combined H + F bright pixels, therefore, represent high-priority targets for follow-up field sampling and drilling.

The spatial distribution of hydroxyl anomalies is not random but broadly follows the NNE–SSW structural trend of the AMB, consistent with the structural control on hydrothermal fluid circulation described by Ghebreab et al. [30]. In Site-1, hydroxyl anomalies are spatially associated with the contact zones between felsic volcanic units and intercalated mafic flows, a setting that is analogous to known VMS deposit positions in the belt. In Site-2, hydroxyl anomalies are more extensive and diffuse, potentially reflecting a broader footprint of hydrothermal alteration around the Debarwa deposit or the greater depth of erosion that exposes more of the alteration envelope at surface.

The clearer expression of alteration and lithologic contrast in Site-2 is attributable to its superior rock exposure and lower urban/lateritic cover; conversely, Site-1’s high urbanization and lateritization reduce spectral contrast and complicate direct lithologic correlation with existing geological maps [48,50]. This is an important limitation: the performance of Sentinel-2 alteration mapping is fundamentally constrained by the degree of rock exposure. In areas with >50% laterite or soil cover, multispectral indices mix the signal of the underlying bedrock alteration with that of the cover material, leading to underestimation of alteration intensity. Future studies should integrate topographic wetness index or regolith thickness estimates to deconvolve cover effects.

### 5.2 Discrimination of gossans from laterites

One of the key challenges and contributions of this study is the discrimination of true VMS gossans from widespread hematitic laterites that are spectrally similar in broadband multispectral data. The Chica-Olma ratio composite proved effective for this discrimination: gossans tend to display distinctive spectral mixtures of both iron oxide anomaly (band 4/2) and subordinate SWIR-hydroxyl signal (band 11/12 and 11/8) due to the partial retention of jarosite and hydroxylated iron phases, while laterites display dominant SWIR signal (hydroxyl from kaolinite in the laterite profile) with a less intense iron oxide signal. In the H + F composite image, confirmed gossan sites appear as white or orange-white pixels (high in both H and F), whereas laterites appear predominantly greenish-yellow (high in H, moderate in F). This discrimination approach is consistent with the findings of Abdelsalam et al. [5] for gossans in northern Eritrea and Gersman et al. [29] for hydrothermally altered rocks in the Danakil Depression.

### 5.3 Effectiveness of Sentinel-2 and PCA-based alteration mapping

The efficacy of the Chica-Olma composite and Crosta-style H/F mapping in highlighting hydroxyl and iron-oxide alteration is consistent with prior applications [10,41]. Our results confirm that Sentinel-2 multispectral bands, when combined with feature-oriented PCA and band-ratio methods, provide a rapid, cost-effective approach to delineate alteration zoning at a reconnaissance scale [47,48,50]. However, unlike studies carried out in regions with minimal surface cover, the performance here varies with surface exposure and anthropogenic cover, underscoring the need to contextualize spectral outputs against field conditions [49,51]. Comparison with the recent multi-sensor approach of Torres Rodríguez & Tapia Guerra [14], who combined Sentinel-2, ASTER, and Landsat-9 to improve mineral discrimination in the Coastal Cordillera of Chile, suggests that the addition of ASTER SWIR data (6 bands between 1.6 and 2.5 μm) would substantially improve mineral identification specificity in the AMB, particularly the discrimination between chlorite, sericite, and kaolinite alteration types. We recommend that future studies incorporate ASTER or PRISMA data alongside Sentinel-2 for more detailed mineralogical mapping.

Recent comparisons with hyperspectral (PRISMA/EnMAP) studies indicate that Sentinel-2 is effective for reconnaissance mapping, but that hyperspectral data substantially improve mineral discrimination where available [51,52]. The approach of Eldosouky, Othman, et al. [34], who integrated multispectral, radar, and magnetic data with a Random Forest algorithm for gold prospectivity mapping in highly weathered basement rocks, represents a promising direction for future work in the AMB. Similarly, the integration of geological controls with remote sensing and magnetic data demonstrates the value of multi-dataset fusion for enhancing prospectivity mapping in complex geological settings [16,34].

### 5.4 Classification accuracy and spectral limitations

High accuracies for gossan, laterite, and major basalt units suggest strong spectral separability for these classes at Sentinel-2 resolution and the chosen band sets. Lower accuracy for pillowed mafic flows and confusion between felsic subunits likely arises from overlapping mineral assemblages (e.g., feldspar, quartz, pyroxene) and sub-pixel heterogeneity [41,50]. The confusion between pillowed mafic flows and some mafic tuff/breccia units (both characterized by plagioclase and pyroxene/amphibole) is expected, as these units have nearly indistinguishable broadband spectral signatures at Sentinel-2 resolution. This ambiguity would be reduced with hyperspectral data capable of resolving fine mineral absorption features in the SWIR. The moderate accuracy for metasediments in Site-2 (Table S2 in S1 File) reflects the spectral heterogeneity of this unit, which includes varied proportions of chlorite, quartz, and carbonaceous material.

These ambiguities emphasize that pixel-based multispectral approaches may misclassify lithologies with similar spectral endmembers; object-based or spectral mixture analyses, or higher spectral resolution data (hyperspectral), could reduce misclassification [51,52]. Petrographic confirmation of alteration minerals (chlorite, epidote, altered plagioclase) supports the spectral assignments from remote sensing and validates the applied H and F extraction rules. Together, these lines of evidence increase confidence in prioritizing targets for exploration.

The multi-method comparison (ML, Min-dist, SAM) reveals important methodological insights. Min-dist, being a simpler algorithm, is computationally efficient and performs well where classes are spectrally distinct, but it tends to overclassify spectrally dominated areas. ML provides the most statistically rigorous classification but is sensitive to training sample size and distribution. SAM performed comparably to ML for spectrally pure classes (gossan, basalt, laterite) but showed more confusion for spectrally mixed classes. The selection of the best-performing classifier for operational use should therefore be site-specific, guided by the accuracy assessment results.

### 5.5 Limitations and recommendations for future research

This study has several limitations that should be acknowledged. First, the temporal coverage is limited to a single Sentinel-2 scene (January 2017), and multi-temporal analysis could potentially improve alteration mapping by exploiting seasonal vegetation differences and reducing cloud-shadow effects. Moreover, the petrographic validation was limited to 14 thin sections, which, while geographically distributed, cannot fully represent the spectral variability of all lithological classes across the two sites.

Future research directions include: (1) integration of ASTER or PRISMA hyperspectral data with Sentinel-2 to improve mineral specificity; (2) application of machine learning approaches (Random Forest, Support Vector Machine, or deep learning) to the full Sentinel-2 band stack and derived indices, following approaches demonstrated by Eldosouky, Othman, et al. [34]; (3) high-resolution airborne geophysical surveys (electromagnetics, magnetics) to constrain the geometry and extent of subsurface sulfide bodies; and (4) geochemical sampling of anomalous gossan targets identified in this study to assess their economic potential.

## 6. Conclusions

The following conclusions are drawn from this study: Sentinel-2 multispectral imagery was applied for the first time systematically to both the northern (Embaderho–Adi Nefas) and southern (Debarwa–Kodadu) segments of the Asmara Mineralized Belt. Gossans, the surface expressions of VMS deposits, were successfully differentiated from spectrally similar but barren hematitic laterites using the Chica-Olma band ratio composite and the FPCS H + F composite, providing a reliable remote sensing criterion for gossan identification in this geological setting.

The combination of band ratio analysis (Chica-Olma, Abrams’ composites), Feature-Oriented Principal Component Selection (FPCS) for hydroxyl (H) and iron-oxide (F) mapping, and supervised classification (ML, Min-dist, SAM) provides a robust, scalable, and cost-effective workflow for reconnaissance-scale mineral mapping in the AMB. The FPCS technique successfully isolated hydroxyl and iron-oxide spectral variance in PC3 and PC4 respectively, consistent with the Guha et al. (2018) and Loughlin [40]methodology. Supervised classification yielded high overall accuracies for gossan, laterite, and major basalt units; lower accuracies for spectrally overlapping mafic sub-units reflect the fundamental limitations of Sentinel-2’s multispectral resolution.

The delineated gossan and alteration zones in the AMB show strong spatial correspondence with known VMS deposit locations, and petrographically confirmed hydrothermal alteration mineral assemblages (chlorite, epidote, sericite). These observations collectively validate the suitability of the proposed remote sensing workflow as a first-pass exploration tool for VMS mineralization in the Arabian-Nubian Shield. The H + F composite targets identified in Site-1 and Site-2 represent high-priority areas for follow-up field investigation, geochemical sampling, and geophysical surveys.

Future studies should: integrate ASTER or PRISMA hyperspectral data to enhance mineral discrimination specificity; apply machine learning algorithms (Random Forest, deep learning) to Sentinel-2 and multi-sensor data stacks for improved prospectivity mapping across the full AMB; conduct high-resolution airborne geophysical surveys to characterize the subsurface geometry of mineralized targets; and undertake systematic geochemical sampling of priority gossan targets identified from this study. The methodology developed here can be applied to other geologically similar Neoproterozoic VMS-hosting terranes within the Arabian-Nubian Shield and elsewhere in sub-Saharan Africa.

## Supporting information

S1 File Supplementary data. (DOCX)
