# Supplementary material for: A data-driven remote sensing approach for VMS mineralization mapping: Integrating Sentinel-2 imagery with geology data in the Asmara Belt, Eritrea
Source: PLoS One. 2026 Jul 24;21(7):e0353934. doi: 10.1371/journal.pone.0353934 (PMC13399357; doi:10.1371/journal.pone.0353934)
Supplement: S1 File — (DOCX) [file pone.0353934.s001.docx]

**A data-driven remote sensing approach to VMS mineralization mapping: Integrating Sentinel-2 imagery with geology data in the Asmara Belt, Eritrea**

Segen M. Habtemichael^1^, Woldegabriel Genzebu^1^, Selamawit T. Ghebremicael^2^, and Yacob T. Tesfaldet^1,*^

^1^Department of Earth Science, Mai-Nefhi College of Science, Eritrea

^2^Alpha Exploration, Asmara, Eritrea

Correspondence email: [yacob.t.tesfaldet@gmail.com](mailto:yacob.t.tesfaldet@gmail.com); ORCID: https://orcid.org/0000-0002-3763-6913

Table S1. Confusion matrix for Min-dist classification on 10 VNIR and SWIR Sentinel-2 bands of Site-1.

| Reference data | | | | | | | | | | | | | | |
| --- | --- | --- | --- | --- | --- | --- | --- | --- | --- | --- | --- | --- | --- | --- |
| Classified data | Class | Vegetation | Built-up | | Water bodies | GRAN | MAFF | | LATR | FELF | MAFU | FLBA | FERU | Total |
|  | Vegetation | **1542** | 0 | | 0 | 0 | 0 | | 0 | 0 | 164 | 0 | 0 | 1706 |
|  | Built-up | 0 | **5514** | | 11 | 0 | 1577 | | 0 | 135 | 100 | 1 | 0 | 7338 |
|  | Water bodies | 0 | 0 | | **1530** | 0 | 9 | | 0 | 42 | 0 | 0 | 0 | 1581 |
|  | GRAN | 0 | 0 | | 0 | **3319** | 1218 | | 0 | 112 | 43 | 0 | 0 | 4692 |
|  | MAFF | 0 | 63 | | 0 | 0 | **19966** | | 0 | 1069 | 3323 | 0 | 0 | 24421 |
|  | LATR | 0 | 0 | | 1 | 0 | 29 | | **3703** | 46 | 74 | 0 | 0 | 3853 |
|  | FELF | 0 | 20 | | 0 | 62 | 3712 | | 33 | **17963** | 5391 | 0 | 0 | 27181 |
|  | MAFU | 187 | 1 | | 0 | 0 | 2531 | | 0 | 2478 | **10510** | 0 | 0 | 15707 |
|  | FLBA | 5 | 5 | | 6 | 4 | 226 | | 3 | 390 | 423 | **834** | 0 | 1896 |
|  | FERU | 0 | 0 | | 0 | 0 | 0 | | 0 | 0 | 118 | 0 | **79** | 197 |
|  | Total | 1734 | 5603 | | 1548 | 3385 | 29268 | | 3739 | 22235 | 20146 | 835 | 79 | **88572** |
|  |  | | | | | | | | | | | | | |
| Class  Vegetation  Built-up  Water bodies  GRAN  MAFF  LATR  FELF  MAFU  FLBA  FERU | | | | Producer’s Accuracy (%)  88.92  98.41  98.84  98.05  68.22  99.04  80.79  52.17  99.88  100.00 | | | | User’s Accuracy (%)  90.39  75.14  96.77  70.74  81.76  96.11  66.09  66.91  43.99  40.10 | | | | | | |
| Overall Accuracy | | | | 73.34 | | | | | | | | | | |
| Kappa Coefficient | | | | 0.66 | | | | | | | | | | |

Table S1. Confusion matrix for ML classification on 10 VNIR and SWIR Sentinel-2 bands of Site-2.

| Reference data | | | | | | | | | | | | | | | | | | | | | |
| --- | --- | --- | --- | --- | --- | --- | --- | --- | --- | --- | --- | --- | --- | --- | --- | --- | --- | --- | --- | --- | --- |
| Classified data | Class | Vegetation | Water bodies | Built-up | FELB | GRAN | | | METS | | LATR | | FELF | | MAFF | | FLBA | SAPR | | Total | |
|  | Vegetation | **3322** | 5 | 2 | 138 | 34 | | | 207 | | 30 | | 2 | | 32 | | 242 | 1 | | 4015 | |
|  | Water bodies | 0 | **2696** | 0 | 0 | 0 | | | 3 | | 0 | | 0 | | 0 | | 3 | 0 | | 2702 | |
|  | Built-up | 0 | 2 | **2847** | 31 | 23 | | | 825 | | 167 | | 0 | | 295 | | 1723 | 0 | | 5913 | |
|  | FELB | 137 | 9 | 34 | **6679** | 154 | | | 1792 | | 521 | | 291 | | 1642 | | 579 | 8 | | 11846 | |
|  | GRAN | 0 | 0 | 0 | 62 | **15031** | | | 1043 | | 1432 | | 124 | | 1596 | | 13 | 0 | | 19301 | |
|  | METS | 46 | 21 | 152 | 964 | 540 | | | **27277** | | 802 | | 181 | | 3199 | | 670 | 11 | | 33863 | |
|  | LATR | 0 | 0 | 20 | 461 | 1410 | | | 756 | | **11381** | | 127 | | 3086 | | 72 | 105 | | 17418 | |
|  | FELF | 15 | 0 | 0 | 51 | 83 | | | 255 | | 116 | | **15318** | | 257 | | 78 | 77 | | 16250 | |
|  | MAFF | 8 | 0 | 19 | 741 | 1420 | | | 5788 | | 1874 | | 96 | | **12511** | | 96 | 60 | | 22613 | |
|  | FLBA | 15 | 5 | 547 | 444 | 163 | | | 1123 | | 111 | | 43 | | 292 | | **15246** | 0 | | 17989 | |
|  | SAPR | 14 | 0 | 9 | 485 | 128 | | | 327 | | 566 | | 208 | | 894 | | 13 | **2118** | | 4762 | |
|  | Total | 3557 | 2738 | 3630 | 10056 | | 18986 | 39396 | | 17000 | | 16390 | | 23804 | | 18735 | | 2380 | | | **156672** |
| \| **Class** \| \| --- \| \| **Vegetation** \| \| **Water bodies** \| \| **Built-up** \| \| **FELB** \| \| **GRAN** \| \| **METS** \| \| **LATR** \| \| **FELF** \| \| **MAFF** \| \| **FLBA** \| \| **SAPR** \| | | | Producer’s Accuracy (%)   \| 93.39 \| \| --- \| \| 98.47 \| \| 78.43 \| \| 66.42 \| \| 79.17 \| \| 69.24 \| \| 66.95 \| \| 93.46 \| \| 52.56 \| \| 81.38 \| \| 88.99 \| | | | | | User’s Accuracy (%)   \| 82.74 \| \| --- \| \| 99.78 \| \| 48.15 \| \| 56.38 \| \| 77.88 \| \| 80.55 \| \| 65.34 \| \| 94.26 \| \| 55.33 \| \| 84.75 \| \| 44.48 \| | | | | | | | | | | |  | | |
| Overall Accuracy | | | 73.04 | | | | | | | | | | | | | | | | | | |
| Kappa Coefficient | | | 0.69 | | | | | | | | | | | | | | | | | | |
